# Enhancing seniors’ dental care access: Analyzing the impact of government insurance in Canada

**DOI:** 10.1371/journal.pone.0310928

**Published:** 2024-09-24

**Authors:** Qi Zhang

**Affiliations:** 1 Institute of Health Policy, Management and Evaluation, University of Toronto, Toronto, ON, Canada; 2 Canadian Centre for Health Economics, Toronto, ON, Canada; Shahid Beheshti University of Medical Sciences School of Dentistry, IRAN, ISLAMIC REPUBLIC OF

## Abstract

A crucial policy question for the government is whether publicly funded insurance programs effectively improve access to care. Using 2015 and 2018 Canadian Community Health Survey (CCHS) data, we first estimated the effect of government dental insurance for seniors on promoting regular care access and lowering cost barrier. When controlling for individual heterogeneity, we found that having government coverage is associated with significantly lower probability of reporting avoidance of dental care due to cost compared to having no coverage. This effect is comparable with other types of insurance. However, the impact of the government program on regular access to dental care is modest. Secondly, using a portion of data collected in Alberta, we found that the government plan does not increase the overall coverage rate. Moreover, switching from an employer-based plan to government-provided coverage for seniors reduces the probability of regular access to care and increases the probability of experiencing cost barrier. This finding indicates that without expansion of overall coverage rate, the current government dental program may not be generous enough to offset the negative impact of leaving the employer-based plan.

## Introduction

Like most wealthy nations, Canada has universal health insurance coverage of physician and hospital-based services [1]. However, dental care expenses are not included in the universal plan, and are instead financed through a combination of employer-sponsored plans, government funded programs, private plans and out of pocket payments. According to Canadian Dental Association, in 2015, public expenditure accounted for only 6.2 percent of total spending, leaving the majority of costs to be covered by the private sector. Within the private sector, approximately 60 percent of expenditures were covered by insurance, while the remaining 40 percent were paid direct out-of pocket [2]. Not all Canadian have dental insurance coverage, and this patchwork system is susceptible to creating inequalities in access to care.

The primary goal of insurance is to reduce financial risk and provide access to care that would otherwise be unaffordable [3]. In the Canadian context, over 60 percent of dental insurance coverage is employer based, while private insurance coverage accounts for about 10 percent [4]. Additionally, most provinces and territories in Canada have provincial government dental programs for low income seniors aged 65 years and above. Many individuals of advanced age may leave their employer-based plan after retirement. Moreover, the decline in income may impose a financial burden, preventing them from purchasing a private plan. Given the dental insurance system in Canada, there are two questions related to elderly individuals that are worth examining carefully. First, what is the effect of government insurance for seniors on the use of dental service compared not only to having no coverage but also to having other types of coverage? Second, to what extent can a change in the type of insurance coverage (e.g., from employ-based to government program for seniors) affect an individual’s care-seeking behavior?

There is a substantial literature related to the first question. Previous studies have found significant positive associations between dental service use and oral health, as well as between dental insurance coverage and dental service utilization [5–8]. In a study that employed a simultaneous equation framework with Australian data, researchers found that having insurance coverage is associated with 56 percentage points higher probability of seeing a dentist, and the predicted probability of visiting a dentist would increase by 43 percentage points if those individuals without coverage could obtain private insurance coverage [9].

In a study conducted in Canada, researchers found that reported insured individuals are approximately 20 percentage points more likely to receive dental care than those without coverage [10]. Several other studies employed Canadian data and logit models to estimate that individual characteristics such as age, immigrant status, education, income, insurance, and self-reported oral health are significantly associated with one’s likelihood of avoiding care due to cost barriers [11–14].

Previous studies typically selected samples of individuals aged 12 and over, focusing on indicators of insurances coverage versus no coverage or broadly classifying coverage into private, public and or uncovered categories. In this study, we take a more detailed approach by meticulously mapping out the specific types of insurance held by aged individuals (65 years old or above). We also account for the possibility that an individual may have coverage through more than one type of insurance. Additionally, we distinguish between individuals who intend to use dental care and those who do not, which is relevant for the selection of samples for analyzing cost barriers. Furthermore, we leverage the shift in insurance coverage mix between the age group 60 to 64 and 65 to 69, to further highlight the impact of the government dental program in comparison to other types of insurance, such as employer-based plan. The primary objective of this study is to evaluate the effect of government dental insurance for seniors on promoting regular access to care and alleviating cost barriers. To the best of our knowledge, this is the first study to attempt to estimate the impact of government dental insurance for seniors not only in comparison to uncovered individuals but also in relation to individuals covered by different types of insurance.

The rest of the paper is structured as follows: it begins with a description of the data, variable specifications, and criteria used to construct samples for regression analysis. Following that, we outline the empirical framework and estimation strategies. Next, we present the main empirical results. Finally, the paper concludes in the last section.

## Data and variable specifications

The data used for this study are derived from the 2015 and 2018 Canadian Community Health survey (CCHS), a federal cross-sectional servery [4]. The survey’s target population comprises all Canadian residents aged 12 years and above. To sample the adult population, a two-stage stratified cluster design was employed, utilizing the same area frame as the Canadian Labor Force Survey. For the youth population, the Canadian Child Tax Benefits file was used as a list frame. The survey contains a wide range of self-reported information including health status, health insurance arrangements, health care utilization, health risk behaviors, and demographic characteristics. Data collection was conducted through telephone interviews or computer-assisted personal interviews. Weights were included in the survey to produce estimates that are representative of the covered population rather than the sample itself. It is important to note that a redesigned version of the survey was implemented in 2015, which imposes restrictions on combining the 2015 data with CCHS data from previous years [15].

The majority of respondents who answered dental-related questions in the 2015 survey were from Alberta, while in the 2018 survey, they were predominantly from Ontario. There are two reasons for not pooling observations from Alberta and Ontario: 1) the government dental program for seniors is organised at the provincial level, each having its own enrollment criteria and reimbursement policy; and 2) public opinion regarding access to dental care may have evolved over the three-year interval. This study focuses on two separate samples: 2,454 individuals from Alberta and 9,065 individuals from Ontario, all aged 65 years and over. After excluding missing observations, we use these samples to estimate the effect of the government program for seniors on accessing dental care.

### The dependent variables

To measure access to care, two binary dependent variables are constructed using various survey questions. Questions related to dental care access are as follows: *How often do you see a dental professional?*(Question 1), *In the past 12 months, have you avoided going to dental professional because of the cost of dental care?* (Question 2), and *Was the last time you saw a dental professional in less than 1 year to 1 year ago?* (Question 3). The constructed “Regular access” variable takes value 1 if the respondent answered Question 1 with “more/about once a year for check-ups or treatment”, and takes value 0 if the response was “less than once a year/only for emergency care/never”.

The other dependent variable “Cost barrier” is constructed using aforementioned Question 2 and Question 3. A “Yes” response to Question 2 indicates the unaffordability of needed dental care in the past 12 month or reduced quantity of dental care use due to cost. Conversely, a “No” response implies two distinct scenarios: 1) the individual sought dental care, and cost was not a barrier; 2) the individual had no intention of seeking dental care in the past 12 months. Since insurance coverage is more relevant to lowering cost barriers for individuals who intend to use care, it is crucial to distinguish these two scenarios. We combine information from Question 3 with Question 2 to make the distinction.

The detailed response patterns on Question 2 and question 3 for respondents from Alberta and Ontario are summarized in Tables 1 and 2, respectively.

**Table 1:** Summary of responses: Age 65+, Alberta.

		Question 3	
		*Yes*	*No*
Question 2	*Yes*	235	198
	*No*	1, 331	691

**Table 2:** Summary of responses: Age 65+, Ontario.

		Question 3	
		*Yes*	*No*
Question 2	*Yes*	862	982
	*No*	5, 511	1, 710

The purpose of dental insurance is to alleviate cost barrier for individuals who would otherwise be unable to afford dental care (i.e, to help move these in the first row of Table 1 to the left cell of the second row). For individuals in the right cell of the second row, it is unlikely that cost barriers are the reason they do not seek care. Since insurance coverage affects them differently from the rest of the sample, we excluded these respondents from our analysis when using “Cost barrier” as the outcome variable. This results in a reduced sample size of 1,767 for respondents from Alberta, and 7,355 for respondents from Ontario, respectively.

### The explanatory variables

The key explanatory variables of interests are the types of insurance held by respondents. The survey includes six different types of dental insurance programs, namely: employer-based plan, government program for seniors, private plan, government program for social services clients, government program for first nations, and other type of insurance. Additionally, some respondents were covered by more than one program. To account for this, a binary variable “multi-insurance” is constructed to take value 1 if a respondent was covered by more than one type of insurance. It is worth nothing that very few studies in the literature have specified the type of insurance the respondents had or have taken into account the possibility of individuals having more than one type of insurance. Tables 3 and 4 report the coverage pattern in Alberta and Ontario, receptively.

**Table 3:** Dental insurance coverage: Age 65+, Alberta.

Employer based	GOVT seniors	Private plan	GOVT social service clients	GOVT first nations	Other insurance	Frequency	%
						*651	*26.53%
X						376	15.32%
	X					905	36.88%
		X				295	12.02%
			X			22	0.90%
				X		8	0.33%
					X	17	0.69%
X	X					81	3.30%
X	X	X				1	0.04%
X	X			X		1	0.04%
X		X				13	0.53%
X					X	3	0.12%
	X	X				69	2.81%
	X		X			3	0.12%
	X			X		1	0.04%
	X				X	3	0.12%
	X	X	X			1	0.04%
		X	X			3	0.12%
		X			X	1	0.04%
						2,454	100%

Note:

*The first row indicates no insurance coverage.

**Table 4:** Dental insurance coverage: Age 65+, Ontario.

Employer based	GOVT seniors	Private plan	GOVT social service clients	GOVT first nations	Other insurance	Frequency	%
						*5,431	*59.91%
X						2,597	28.65%
	X					247	2.72%
		X				594	6.55%
			X			39	0.43%
				X		29	0.32%
					X	37	0.41%
X	X					30	0.33%
X	X	X				1	0.01%
X	X		X			2	0.02%
X	X			X		1	0.01%
X		X				27	0.30%
X			X			1	0.01%
X				X		6	0.07%
X					X	5	0.06%
	X	X				7	0.08%
	X		X			2	0.02%
	X			X		1	0.01%
	X				X	2	0.02%
		X	X			3	0.03%
		X		X		1	0.01%
		X			X	1	0.01%
			X		X	1	0.01%
						9,065	100%

Note:

*The first row indicates no insurance coverage.

In Alberta and Ontario, the majority of aged adults were covered by a single insurance program, either an employer-sponsored plan, the government plan for seniors, or a private plan. In Alberta, only about 26.5 percent aged adults had no dental insurance coverage, which is lower than the 59.9 percent in Ontario. Approximately 15 percent of Albertans aged 65 years or over were covered by the employer-based plan, compared to 29 percent with employer-based coverage in Ontario. However, in Ontario, only 2.7 percent aged individuals were covered by the government program for seniors, whereas the counterpart program in Alberta had a coverage of 36.9 percent. This difference is largely due to the more generous eligibility criteria for enrollment in the program in Alberta. For instance, the annual income thresholds for eligibility in the Ontario program was CAD 19,300 for single seniors and CAD 32,300 for senior couples, while the thresholds in Alberta were higher, at CAD 31,675 for single seniors and CAD 63,350 for senior couples. Moreover, the private plan coverage seemed to be more prevalent in Alberta.

In terms of other explanatory variables, the specifications in CCHS are largely consistent with those in Canadian Health Measures Survey (CHMS) and National Population Health Survey (NPHS), which were the main data sources of previous work used Canadian data. In the literature, demographic controls typically include: age (as one of the 4 bands; 1 is 64 to 69 years old, and 4 is 80 years old or more), sex, whether the individual was born in Canada, whether the individual completed post-secondary education, household gross income (as one of the five bands, band width $20,000; from less than $20,000 to $80,000 and above), marital status (as one of the three, married or common law; separated, divorced or widowed, single), and dwelling status (owned or rented) [10, 11]. In addition, we also controlled for the size of household, which was categorized into five bands (1, 2, 3, 4, and 5 or above).

### Descriptive statistics and variable definitions

Table 5 presents the definitions and summary statistics for all the explanatory variables along with “Regular access” used as dependent variable. Similar information with “Cost barrier” used as dependent variable are shown in Table 6.

**Table 5:** Summary statistics and variable definitions-regular access sample.

Variable	Description	Alberta Mean	Ontario Mean
*Outcome Variable*			
Regular access	= 1 if answered less than or about once a year to question 1; = 0 otherwise	0.634	0.694
*Explanatory Variable*			
employer	= 1 if in an employer-sponsored plan; = 0 otherwise	0.194	0.295
govt-seniors	= 1 if in a provincial government program for seniors; = 0 otherwise	0.434	0.032
private	= 1 if in a private plan; = 0 otherwise	0.156	0.070
govt-social	= 1 if in a government program for social service clients; = 0 otherwise	0.012	0.005
govt-fn	= 1 if in a government program for First Nations; = 0 otherwise	0.004	0.004
other	= 1 in in a other type of dental insurance program; = 0 otherwise	0.010	0.005
multi-insurance	= 1 if covered by two or more programs; = 0 otherwise	0.073	0.010
female	= 1 if female; = 0 otherwise	0.566	0.578
Age			
age65-69	= 1 if age between 65 and 69; = 0 otherwise	0.353	0.331
age70-74	= 1 if age between 70 and 74; = 0 otherwise	0.256	0.260
age75-79	= 1 if age between 75 and 79; = 0 otherwise	0.183	0.179
age80+	= 1 if age 80 and older; = 0 otherwise	0.208	0.230
postsec	= 1 if completed post-secondary education; = 0 otherwise	0.524	0.503
canada	= 1 if born in Canada; = 0 otherwise	0.789	0.742
dwell	= 1 if owned dwelling; = 0 otherwise	0.831	0.778
Marital Status			
marital1	= 1 if married or common-law; = 0 otherwise	0.541	0.532
marital2	= 1 if widowed, separated or divorced; = 0 otherwise	.411	0.407
marital3	= 1 if single; = 0 otherwise	0.048	0.061
Household Income			
hincome1	= 1 if <$20k; = 0 otherwise	0.074	0.074
hincome2	= 1 if $20k to < $40k; = 0 otherwise	0.283	0.245
hincome3	= 1 if $40k to < $60k; = 0 otherwise	0.206	0.211
hincome4	= 1 if $60k to < $80k; = 0 otherwise	0.141	0.151
hincome5	= 1 if ≥$80k; = 0 otherwise	0.296	0.319
Household Size			
hsize1	= 1 if 1 person; = 0 otherwise	0.427	0.430
hsize2	= 1 if 2 persons; = 0 otherwise	0.513	0.502
hsize3	= 1 if 3 persons; = 0 otherwise	0.039	0.045
hsize4	= 1 if 4 persons; = 0 otherwise	0.013	0.013
hsize5	= 1 if 5 or more persons; = 0 otherwise	0.008	0.010
N		2,454	9,065

**Table 6:** Summary statistics and variable definitions-cost barrier sample.

Variable	Description	Alberta Mean	Ontario Mean
*Outcome Variable*			
Cost barrier	= 1 if answered “Yes” to question 2; = 0 otherwise	0.245	0.251
*Explanatory Variables*			
employer	= 1 if in an employer-sponsored plan; = 0 otherwise	0.222	0.312
govt-seniors	= 1 if in a provincial government program for seniors; = 0 otherwise	0.391	0.029
private	= 1 if in a private plan; = 0 otherwise	0.176	0.078
govt-social	= 1 if in a government program for social service clients; = 0 otherwise	0.011	0.005
govt-fn	= 1 if in a government program for First Nations; = 0 otherwise	0.003	0.004
other	= 1 in in a other type of dental insurance program; = 0 otherwise	0.011	0.005
multi-insurance	= 1 if covered by two or more programs; = 0 otherwise	0.082	0.010
female	= 1 if female; = 0 otherwise	0.573	0.590
Age			
age65-69	= 1 if age between 65 and 69; = 0 otherwise	0.399	0.355
age70-74	= 1 if age between 70 and 74; = 0 otherwise	0.250	0.267
age75-79	= 1 if age between 75 and 79; = 0 otherwise	0.167	0.176
age80+	= 1 if age 80 and older; = 0 otherwise	0.184	0.202
postsec	= 1 if completed post-secondary education; = 0 otherwise	0.587	0.542
canada	= 1 if born in Canada; = 0 otherwise	0.776	0.737
dwell	= 1 if owned dwelling; = 0 otherwise	0.858	0.802
Marital Status			
marital1	= 1 if married or common-law; = 0 otherwise	0.574	0.552
marital2	= 1 if widowed, separated or divorced; = 0 otherwise	.379	0.387
marital3	= 1 if single; = 0 otherwise	0.047	0.061
Household Income			
hincome1	= 1 if <$20k; = 0 otherwise	0.052	0.066
hincome2	= 1 if $20k to < $40k; = 0 otherwise	0.249	0.222
hincome3	= 1 if $40k to < $60k; = 0 otherwise	0.217	0.205
hincome4	= 1 if $60k to < $80k; = 0 otherwise	0.148	0.158
hincome5	= 1 if ≥$80k; = 0 otherwise	0.333	0.349
Household Size			
hsize1	= 1 if 1 person; = 0 otherwise	0.402	0.412
hsize2	= 1 if 2 persons; = 0 otherwise	0.539	0.521
hsize3	= 1 if 3 persons; = 0 otherwise	0.039	0.045
hsize4	= 1 if 4 persons; = 0 otherwise	0.013	0.013
hsize5	= 1 if 5 or more persons; = 0 otherwise	0.009	0.009
N		1,764	7,355

### The government dental program for seniors and coverage-mix shift, a case in Alberta

According to the 2015 Environmental Scan of Publicly Financed Dental Care in Canada, the public dental program in Alberta has a reputation for providing benefits to seniors. Since 2004, consistent tax dollars have been invested in the government funded plan [16]. Given the well-established program in Alberta, we turn our attention to Albertans in the age group of 60 to 64 years and the age group 65 to 69 years. Unfortunately, conducting a similar analysis with data from Ontario is not feasible due to the low number of Ontarian in the 65 to 69 years age group with coverage from government plan for seniors. Individuals from age group 60 to 64 years and age group 65 to 69 years are expected to have similar demand and attitude towards accessing dental care. Therefore, the differences observed in “Regular access” and “Cost barrier” are likely to be attributed to the shift of insurance type. The public program may provide certain coverage for seniors over 65 years old who would otherwise remain uninsured. Meanwhile, seniors over 65 years old are likely to leave the employer sponsored plan due to retirement. As a result, the overall picture is remains. To fully illustrate these points, Tables 7 and 8 reports the insurance coverage pattern for these two age groups.

**Table 7:** Dental insurance coverage: Age 60-64, Alberta.

Employer based	GOVT seniors	Private plan	GOVT social service clients	GOVT first nations	Other insurance	Frequency	%
						*228	*25.28%
X						446	49.45%
		X				126	13.97%
			X			54	5.99%
				X		3	0.33%
					X	25	2.77%
X		X				12	1.33%
X			X			1	0.11%
X				X		1	0.11%
X					X	2	0.22%
		X	X			1	0.11%
		X			X	2	0.22%
			X		X	1	0.11%
						902	100%

Note:

*The first row indicates no insurance coverage.

**Table 8:** Dental insurance coverage: Age 65-69, Alberta.

Employer based	GOVT seniors	Private plan	GOVT social service clients	GOVT first nations	Other insurance	Frequency	%
						*213	*24.62%
X						213	24.62%
	X					233	26.94%
		X				115	13.29%
			X			5	0.58%
				X		2	0.23%
					X	7	0.81%
X	X					39	4.51%
X	X			X		1	0.04%
X		X				9	1.04%
X					X	3	0.12%
	X	X				23	2.66%
	X		X			1	0.12%
		X			X	1	0.12%
						865	100%

Note:

*The first row indicates no insurance coverage.

It is worth noting that the overall coverage rate is approximately the same between the two adjacent age groups. At the group level, the impact of the government program for seniors on overall coverage is similar as to transfer some individuals who had the employer sponsored plan to the government plan after they turn 65 years old. The effect of coverage shift from employer-sponsored plans to government plan for seniors among the covered individuals has not been investigated before. The government program is usually less generous compared to employer- sponsored plans. Without expanding overall coverage, such a shift of in the coverage mix could have a negative effect on accessing dental care among individuals with dental insurance coverage. More specifically, switching to the government program for seniors is likely to decrease the probability of regular access of dental care or increase the probability of experiencing a cost barrier. Table 9 presents the summary statistics of the insurance covered individuals age 60 to 64 and age 65 to 69, for samples with dependent variable “Regular access” and “Cost barrier”.

**Table 9:** Summary statistics of age 60-64 and age 65-69 with insurance coverage, Alberta.

Variable	Regular access sample			Cost barrier sample		
	Age 60-64	Age 65-69	p-value	Age 60-64	Age 65-69	p-value
regular access	0.797	0.747	0.031			
cost barrier				0.177	0.213	0.121
govt-seniors	0	0.456	NA	0	0.419	NA
multi-insurance	0.030	0.118	<0.001	0.033	0.120	<0.001
female	0.534	0.499	0.194	0.552	0.514	0.233
age65-69	0	1	NA	0	1	NA
postsec	0.665	0.0.626	0.139	0.691	0.65	0.177
canada	0.852	0.788	0.003	0.854	0.777	0.002
dwell	0.868	0.857	0.576	0.881	0.859	0.298
Marital Status			0.029			0.015
marital1	0.645	0.658		0.646	0.667	
marital2	0.257	0.287		0.251	0.282	
marital3	0.098	0.055		0.103	0.051	
Household Income			<0.001			<0.001
hincome1	0.065	0.064		0.067	0.051	
hincome2	0.105	0.186		0.094	0.173	
hincome3	0.145	0.169		0.157	0.166	
hincome4	0.123	0.147		0.125	0.149	
hincome5	0.561	0.434		0.577	0.461	
Household Size			0.505			0.433
hsize1	0.331	0.316		0.331	0.309	
hsize2	0.573	0.595		0.579	0.598	
hsize3	0.059	0.064		0.051	0.065	
hsize4	0.022	0.011		0.024	0.011	
hsize5	0.015	0.014		0.016	0.017	
N	674	652		553	525	

Note: The Pearson chi-square test was used to compare all variables between the two groups based on frequencies of each category.

## Estimation strategy

For both the Alberta and Ontario samples, linear probability models are estimated as the following:

$$\begin{array}{c} \text{Pr}(Y_{i}=1|I_{i},X_{i})=I_{i}\gamma +X_{i}\beta +\delta_{i}, \end{array}$$ (1)

where i indexes individuals, *Y*_*i*_ is an indicator variable for “Regular access” or “Cost barrier”, *I*_*i*_ is a vector of the six insurance type indicators and the “multi-insurance” indicator, and *X*_*i*_ is a vector of individual controls. The coefficients associated with “govt-seniors” is the effect of the government program for seniors relative to individuals without dental coverage.

### Coverage-mix shift models, Alberta

To determine the effect of government dental program through the coverage mix shift, linear probability models are estimated analogous to the model in Eq (1) with adults aged 60 to 69 with dental insurance coverage from Alberta:

$$\begin{array}{c} \text{Pr}(Y_{c}=1|govtSr_{c},multiINS_{c},X_{c})=\gamma_{1}govtSr_{c}+\gamma_{2}multiINS_{c}+X_{c}\beta +\delta_{c}, \end{array}$$ (2)

where c indexes individuals with dental insurance coverage, *Y*_*i*_ is the outcome variable of “Regular access” or “Cost barrier”, *govtSr* is the insurance type indicator “govt-seniors”, *multiINS* is the “multi-insurance” indicator, and *X*_*i*_ is a vector of individual controls.

Taken together evidence from Tables 8 and 9, the model in Eq (2) assumes that the effects of the coverage-mix shift are captured by the increased shared of the government dental plan for seniors, while holding all other variables in the model constant. Since the overall coverage rate is unchanged between the adults aged 60 to 64 and 65 to 69, the government program for seniors essentially serves as a substitute for other types of insurance. This specification allows us to further evaluate the government dental program in comparison to other insurance types, especially the employer-sponsored plans.

## Results

The estimation results for the dependent variables “Regular access” and “Cost barrier” are presented in Tables 10 and 11, respectively.

**Table 10:** Effect of dental insurance coverage on promoting regular access of dental care, age 65+.

	Ontario		Alberta	
	Estimate	Std. Error	Estimate	Std. Error
employers	0.151***	0.010	0.181***	0.027
govt-seniors	0.041	0.028	0.051**	0.024
private	0.176***	0.015	0.190***	0.029
govt-social	0.032	0.068	-0.076	0.091
govt-fn	0.176***	0.065	0.122	0.136
other	0.033	0.063	0.131	0.081
multi-insurance	-0.162***	0.042	-0.059	0.043
female	0.098***	0.009	0.100***	0.019
postsec	0.106***	0.009	0.156***	0.019
canada	-0.024**	0.010	-0.077	0.022
dwell	0.124***	0.013	0.063**	0.028
Age	age65-69 as ref.			
age70-74	-0.010	0.011	-0.050**	0.023
age75-79	-0.019	0.013	-0.061**	0.027
age80+	-0.046***	0.013	-0.045*	0.027
Marital Status	marital1 as ref.			
marital2	-0.089***	0.020	-0.055	0.037
marital3	-0.026	0.025	-0.056	0.051
Household Income	hincome1 as ref.			
hincome2	0.102***	0.022	0.080**	0.040
hincome3	0.179***	0.023	0.217***	0.043
hincome4	0.244***	0.024	0.261***	0.046
hincome5	0.285***	0.023	0.299***	0.045
Household Size	hsize1 as ref.			
hsize2	-0.078***	0.020	-0.063*	0.036
hsize3	-0.135***	0.027	-0.180***	0.055
hsize4	-0.188***	0.044	-0.071	0.076
hsize5	-0.226***	0.051	-0.082	0.097
Constant	0.364***	0.030	0.330***	0.062
N	9065		2454	
R-squared	0.158		0.156	

Note: Heteroscedastic-robust standard errors are presented under Std. Error.

*Significant at 10%,

**significant at 5%,

***significant at 1%.

**Table 11:** Effect of dental insurance coverage on lowering cost barrier of dental care, age 65+.

	Ontario		Alberta	
	Estimate	Std. Error	Estimate	Std. Error
employers	-0.238***	0.009	-0.286***	0.028
govt-seniors	-0.178***	0.026	-0.158***	0.029
private	-0.222***	0.014	-0.206***	0.031
govt-social	-0.229***	0.058	-0.127	0.106
govt-fn	-0.376***	0.039	-0.289*	0.152
other	-0.224***	0.049	-0.176**	0.086
multi-insurance	0.206***	0.030	0.131***	0.041
female	-0.026***	0.009	-0.004	0.021
postsec	-0.033***	0.010	-0.012	0.021
canada	-0.021**	0.010	0.001	0.023
dwell	-0.105***	0.014	-0.062*	0.033
Age	age65-69 as ref.			
age70-74	-0.024**	0.011	-0.055**	0.026
age75-79	-0.060***	0.013	-0.113***	0.029
age80+	-0.135***	0.013	-0.177***	0.029
Marital Status	marital1 as ref.			
marital2	0.082***	0.021	0.044	0.044
marital3	-0.005	0.027	-0.025	0.060
Household Income	hincome1 as ref.			
hincome2	-0.141***	0.025	-0.065	0.055
hincome3	-0.220***	0.026	-0.145**	0.057
hincome4	-0.278***	0.027	-0.187***	0.060
hincome5	-0.323***	0.026	-0.247***	0.058
Household Size	hsize1 as ref.			
hsize2	0.092***	0.021	0.048	0.044
hsize3	0.163***	0.030	0.159**	0.065
hsize4	0.211***	0.047	0.090	0.091
hsize5	0.256***	0.058	0.174	0.110
Constant	0.666***	0.033	0.635***	0.075
N	7355		1764	
R-squared	0.188		0.119	

Note: Heteroscedastic-robust standard errors are presented under Std. Error.

*Significant at 10%,

**significant at 5%,

***significant at 1%.

In Alberta, enrollment in the government plan for seniors is associated with a 5.1 percent higher probability of regular access to dental care, compared to individuals with no insurance coverage. However, similar evidence is not observed among respondents enrolled in the public program for seniors in Ontario. In contrast, for respondents in both provinces, employer-sponsored plans and private plans have a much more pronounced effect on increasing the probability of regular access to care are much more salient.

In comparison with uncovered individuals, enrollment in pubic plan for seniors is associated with 18 percent lower probability of experiencing cost barriers in Ontario and a 16 percent lower probability of experiencing cost barriers in Alberta. Enrollment in employer-based plans and private plans reduces the probability of facing cost barriers by between 21 percent and 29 percent. Not surprisingly, the impact of the public plan for seniors appears to be a smaller magnitude when compared to the employer-sponsored plans and private plans. The government program for seniors seems to be more effective at lowering cost barriers than promoting regular access. This finding may indicate that the government plan for seniors is relatively effective on alleviating cost burden, but may be more restrictive in terms of the types of services covered and frequency limitations.

On average, individuals with higher family incomes are more likely to access dental care regularly and less likely to experience unaffordability in dental care. Individuals with smaller family size are more likely to access dental care regularly and less likely to experience unaffordability of dental care. It is plausible that individuals with more education are more likely to access dental care regularly and less likely to avoid care because of cost, after controlling for household income. More educated individuals enjoy a higher rate of return on a given stock of health, and on average choose a higher optimal stock of health [17]. Owning a residence is an important indicator of socioeconomic status, and is associated with better access to care. Furthermore, the marginal benefit of additional insurance coverage is limited among the covered individuals.

### Coverage-mix shift results

As discussed earlier, the government plan for seniors does not increase the coverage rate in the adults group 65 to 69 years old in relative with the group of 60 to 64 years old. All else being equal, the effects on “Regular access” and “Cost barrier” are mainly caused by shifting into the public program for seniors from other types of insurance. The results for the coverage-mix shift estimation among the covered Albertans are presented in Table 12.

**Table 12:** Effect of insurance coverage change among covered Albertans age 60-64 and age 65-69.

	Regular access		Cost barrier	
	Estimate	Std. Error	Estimate	Std. Error
govt-seniors	-0.108***	0.036	0.121***	0.040
multi-insurance	0.058	0.461	-0.129***	0.045
female	0.092	0.023	0.032	0.024
age65-69	0.020	0.025	-0.022	0.026
postsec	0.150***	0.025	-0.046*	0.027
canada	-0.043	0.028	0.008	0.030
dwell	0.085**	0.040	-0.073	0.046
Marital Status	marital1 as ref.			
marital2	-0.022	0.053	0.093	0.062
marital3	0.034	0.063	0.017	0.067
Household Income	hincome1 as ref.			
hincome2	0.013	0.063	0.035	0.073
hincome3	0.128**	0.061	-0.074	0.070
hincome4	0.139**	0.065	-0.111	0.072
hincome5	0.166***	0.061	-0.160**	0.068
Household Size	hsize1 as ref.			
hsize2	-0.013	0.054	0.073	0.061
hsize3	-0.021	0.069	0.130*	0.075
hsize4	0.139**	0.071	0.129	0.117
hsize5	0.121	0.074	0.077	0.105
Constant	0.488***	0.086	0.282***	0.091
N	1326		1078	
R-squared	0.106		0.091	

Note: Heteroscedastic-robust standard errors are presented under Std. Error.

*Significant at 10%,

**significant at 5%,

***significant at 1%

Among the individuals aged 60 to 69 years with dental insurance coverage, having public dental insurance for seniors is negatively correlated with regular access to care and is positively correlated with experiencing cost barrier when seeking care. On average, switching from employer sponsored plans into the government plan for seniors is expected to result in a nearly 11 percent lower probability of accessing care regularly and about 12 percent higher probability of avoiding care due to cost.

## Conclusion and discussion

This paper examines the effect of government dental insurance for advanced-age individuals in Alberta and Ontario. Using individual level data from 2015 and 2018 CCHS, and employing a linear probability model, we find that enrollment in the government program is associated with a higher probability of regular access of using dental care and a lower the probability of experiencing cost barriers. Relative to the uninsured individuals, the effect magnitude on cost barrier is larger than that on regular access. In Alberta, enrollment in government program is associated with 15.8 percent lower probability of facing cost barriers and 5.1 percent higher probability of regular access to care. Enrollment in the Ontarian program for seniors is associated with a 17.8 percent lower probability of not seeking care due to cost barriers, but there is no evidence that the Ontario program has a significant impact on promoting regular access to dental care.

The government program for seniors in Canada have been successful in alleviating affordability as a major barrier to visiting dental professionals for oral health maintenance and treatment. However, the coverage rates of such programs remain relatively low among aged Canadians. In the sample, approximately 60 percent aged Ontarians were uncovered, while the provincial program for seniors only cover 2.7 percent individuals. An earlier study conducted in Ontario found that having insurance is associated with improved dental visiting behaviors and better oral health status [18]. Even the more generous program in Alberta did not increase the overall coverage rate, when comparing the age group 60 to 64 with age group 65 to 69.

To expand coverage, the income threshold that determines eligibility for enrollment may be worth scrutinizing more carefully by the provincial government. Otherwise, the positive impact of the provincial government dental program for seniors may not be significant enough to offset the negative impact of some seniors leaving their employer-based plans due to retirement. These seniors are likely to face more severe financial barriers to accessing dental care than when they were covered by the employer-based plans. In addition to expanding coverage for seniors, the provincial government could also consider providing more generous dental programs. The types of services on the covered list and the frequency limits deserve closer examination.

This study has several limitations. Firstly, it relies on cross-sectional data, which limits our ability to establish causality. The findings primarily indicate associations, and caution is warranted when interpreting them. Future research could benefit from using panel data to gain a more comprehensive understanding of the long-term effects of the government program for seniors. Secondly, some of the categorical controls employed in this study may not fully capture the nuances of the variables under consideration. For example, the highest income category, capped at CAD 80,000 per year, may not provide sufficient granularity to distinguish differences among higher income earners. This limitation could potentially affect the precision of our findings. Additionally, we acknowledge that assuming uniform demand between the age groups of 60 to 64 and 65 to 69 may oversimplify the complex dynamics at play. Unobservable factors in the dataset, such as individual health behaviors influenced by employment status or industry standards, could introduce biases. For instance, employed individuals may exhibit greater oral health consciousness due to their work environment or industry standards, potentially impacting their dental care utilization. Similarly, the social aspects associated with maintaining good oral health, particularly in the workforce, could lead to variations in dental care behavior that are not fully accounted for in this analysis.

Additionally, in the 2018 CCHS data, only a small portion of seniors 65 years or older in Ontario were covered by government insurance, which may have undermined the robustness of the estimations on the effects of government-sponsored plans from the regression analysis. In January 2020, a new dental plan for seniors in Ontario, called the Ontario Seniors Dental Care Program (OSDCP), was launched. Future studies with more recent data could evaluate the effects of this newly founded program for seniors and compare it with similar programs in other provinces, such as the one in Alberta.

These limitations underscore the need for continued research to refine our understanding of the relationships between dental care utilization, government programs, and individual characteristics, while considering more detailed data, nuanced control variables, and the influence of unobservable factors.
